# ADAMTS-7 Exhibits Elevated Expression in Cartilage of Osteonecrosis of Femoral Head and Has a Positive Correlation with TNF-***α*** and NF-***κ***B P65

**DOI:** 10.1155/2015/196702

**Published:** 2015-01-14

**Authors:** Jing-kun Li, Lei Cheng, Yun-peng Zhao, Ying-jun Guo, Yi Liu, Wei Zhang, Shuai-shuai Wang, Yuan-qiang Zhang, Xin Pan, Lin Nie

**Affiliations:** ^1^Department of Orthopaedic Surgery, Shandong University Qilu Hospital, Jinan, Shandong 250012, China; ^2^Medical College of Shandong University, Jinan, Shandong 250012, China

## Abstract

ADAMTS-7 has been reported to exaggerate cartilage degeneration and to be associated with TNF-*α* and NF-*κ*B signaling pathway. In this study we compared the expression of ADAMTS-7, TNF-*α*, and Phospho-NF-*κ*B in patients with femoral neck fracture (FNF) and osteonecrosis of femoral head (ONFH) at different stages. We found that expression of ADAMTS-7, TNF-*α*, and Phospho-NF-*κ*B was significantly upregulated in ONFH patients' articular cartilage and related to the pathogenesis of ONFH. Thus we conclude that ADAMTS-7 level appears to be positively associated with expression of TNF-*α* and Phospho-NF-*κ*B P65 in cartilage, which may imply its association with cartilage destruction of ONFH.

## 1. Introduction

Osteonecrosis of the femoral head (ONFH) is a common disease in orthopedics throughout the world. Various accepted risks factors, including trauma, excessive steroid use, alcoholism, damage from radiation, sickle cell anemia, Gaucher's disease, and the exposure to high pressures, are reported [1–7]. Chronic groin pain and gait disturbance is one feature of this disease, which is considered to be caused by the secondary osteoarthritis followed by ONFH [8]. Furthermore, when the disease develops to the terminal stage with femoral head collapse and secondary osteoarthritis, a total hip replacement operation is required. To date, the pathogenesis process of ONFH is still unclear [9]. It has been demonstrated that ONFH is caused by the inadequate blood supply of subchondral bone [10–15]. The pathologic process involved in ONFH starts with necrosis in femoral head bone tissue which is thought to be a result of the blood circulation disturbance to the femoral head [16]. During the pathogenesis, the levels of various biomarkers in the femoral head bone, cartilage, synovium, and hip joint synovial fluid are changed, which make effects on the disease process. It has been proved that, in the bone matrix of femoral head in patients with ONFH, expression level of matrix metalloproteinase-2 (MMP-2) is upregulated, which is related to the reduced repair capacity and altered bone remodeling [17]. In the children's ONFH, an increase of proteoglycan fragments in the joint fluid was observed, which implied increased degradation proteoglycan and release of fragments from hip joint cartilage [18]. Moreover, concentrations of proteoglycan fragments, C-propeptide of type-I1 collagen, MMP-3, TIMP-1, and the molar ratios of MMP-3/TTMP-1 are all increased in hip joint fluid of adult ONFH patients even before any evident radiologic changes in the femoral head or hip joint were observed [8]. Collectively, it appears that femoral head cartilage undergoes abnormal metabolic change before collapse in ONFH. However, the pathology of cartilage degradation in ONFH still remains to be elucidated.

The ADAMTS (a disintegrin and metalloproteinase with thrombospondin motifs) family consists of 19 secreted zinc metalloproteinases with a precisely ordered modular organization including at least one thrombospondin type I repeat [19]. To our knowledge, these enzymes have important effects in development, angiogenesis, and coagulation and have relationship with coagulation disorders, malignancy, lumbar disc herniation, rheumatoid arthritis, and osteoarthritis [20–26]. ADAMTS-7, a member of the ADAMTS family, is widely expressed in various tissues. ADAMTS-7 has been reported to negatively regulate endplate cartilage differentiation, facilitate intimal hyperplasia, and mediate vascular remodeling as a novel locus for atherosclerosis [26]. Furthermore, it has been found to degrade cartilage oligomeric matrix protein (COMP) which is an important noncollagenous component of cartilage and may play a key role in osteoarthritis pathogenesis [27–30]. It is overexpressed in cartilage and synovium of arthritis patients, and the level of fragments of COMP in patients' serum is increased concurrently [28]. In vitro, ADAMTS-7 was involved in COMP degradation induced by TNF-*α* [30]. It could negatively mediate chondrocyte differentiation in cartilage differentiation by being a target of parathyroid hormone-related protein (PTHrP) [29, 31]. Moreover, ADAMTS-7 is reported to be closely associated with TNF-*α* and NF-*κ*B signaling pathway, as TNF-*α* mediated NF-*κ*B activation facilitates expression of ADAMTS-7 [31].

The aim of this study was to investigate the expression of ADAMTS-7 in articular cartilage of ONFH patients, to elucidate whether the expression was altered with cartilage degradation and radiological change, and to investigate the potential mechanisms involved, which may indicate new clinic therapeutic interventions for ONFH.

## 2. Materials and Methods

### 2.1. Patients and Tissue Selection

Ethical approval for this study was obtained from the Medical Ethics Committee of Qilu Hospital, Shandong University. What is more, written informed consent from each patient included in this study was obtained.

**Figure 1:**
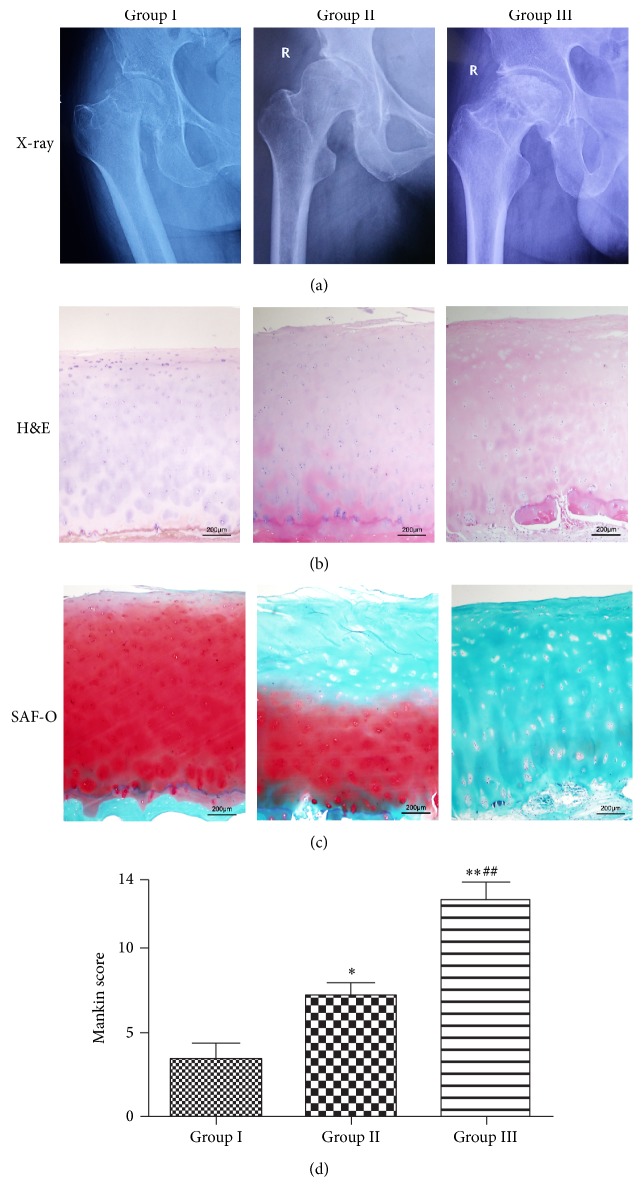
Articular cartilage is degraded in ONFH patients, and the degradation is associated with the pathogenesis of ONFH. (a) Representative X-ray images of three groups. (b) H&E staining of femur articular cartilage. Samples from femur articular cartilage of FNF patients and ONFH patients at different stages, as indicated, were harvested. (c) Safranin-O staining of femur articular cartilage. The cartilage staining was reduced with the progression of ONFH. Representation sections are shown. (d) Mankin score of femur articular cartilage from each group. Group II and group III got statistically significant higher score than group I (^*^ *P* < 0.05, ^**^ *P* < 0.01). Group III got statistically significant higher scores than group II (^#^ *P* < 0.05, ^##^ *P* < 0.01).

Patients and healthy controls with primary osteoarthritis, ankylosing spondylitis (AS), systemic lupus erythematosus (SLE), and acute or chronic inflammatory diseases were excluded. All the patients had taken hip X-ray and magnetic resonance imaging (MRI) examination. The representative X-ray images are shown in Figure 1(a). Human hip articular cartilage specimens were collected from patients with femoral neck fracture (FNF) without ONFH and OA in X-ray as healthy control named group I (*n* = 14; the average age was 64 years with a range of 48 to 85 years) and patients with ONFH (*n* = 34; the average age was 51 years with a range of 41 to 70 years). According to ARCO classification standard [32], articular cartilage specimens obtained from the ONFH patients were categorized into two groups: group II, 15 cases, stage II-III with no obvious collapse in X-ray; Group III, 19 cases, stage IV with obvious femoral head collapse in X-ray. The articular cartilage tissues were dissected carefully from the weight-bearing region of femoral head and subsequently treated according to the corresponding downstream experiments.

### 2.2. Histomorphological Analysis

The cartilage samples were dissected along the axial plane into pieces of 10 mm ∗ 5 mm ∗ 7 mm with a thin layer of subchondral bone and were fixed in 4% formaldehyde for over 24 hours in room temperature. After being decalcified in 10% ethylene diamine tetraacetic acid (EDTA) solution for over 2 weeks, the samples were embedded in paraffin. The cartilage specimens were cut longitudinally into 4 *μ*m thick sections and stained with haematoxylin-eosin (H&E) and safranin-O. We used the Mankin score [33] (Table 1) to evaluate the cartilage degradation.

Immunohistochemistry was also performed. We use primary antibodies for ADAMTS-7 (1 : 200; Abcam Biotechnology Co., Ltd., London, UK), TNF-*α* (1 : 200, Beyotime Institute of Biotechnology, Shanghai, China), and Phospho-NF-*κ*B P65 (1 : 100, Bioss Institute of Biotechnology, Beijing, China). A goat anti-rabbit immunoglobulin- (IgG-) horseradish peroxidase (HRP) secondary antibody (1 : 200; Beijing Golden Bridge Biotechnology Co., Ltd., Beijing, China) was applied. Images were captured by a Nikon Eclipse 80i microscope (Nikon, Tokyo, Japan). Image-Pro Plus software (Media Cybernetics) was used to qualify the average optical density of the ADAMTS-7 positive, TNF-*α* positive, and Phospho-NF-*κ*B P65 positive areas at 400x magnification.

### 2.3. RNA Extraction and Real-Time Quantitative PCR for ADAMTS-7 Gene Expression

Tissue samples were washed with cold PBS and conserved in liquid nitrogen. 2-3 grams of samples was put into liquid nitrogen and tripsised into powder. Total RNA was extracted using TRIzol (Takara Biotechnology Co., Ltd., Dalian, China) method according to the manufacturer's instructions. The quantity and integrity of RNA were verified. Total RNA (1 *μ*g) was reverse-transcribed in a total volume of 10 *μ*L containing 2 *μ*L 5X RT buffer (Toyobo Co., Ltd., Japan), 0.5 *μ*L RT enzyme mix (Toyobo Co., Ltd., Japan), and 0.5 *μ*L prime mix (Toyobo Co., Ltd., Japan). The reaction was performed at 37°C for 15 min and 95°C for 5 min. The resulting cDNA was stored at −80°C until further use. RT-PCR reactions were carried out in triplicate with 100 ng cDNA (RNA equivalent) each with SYBR Green Dye I master mix using a Roche LightCycler (Roche, Switzerland) and the data were analyzed by LightCycler software 4.0.0.23. To normalize ADAMTS-7 gene expression, GADPH was used as an internal control. The genome-wide specificity of the primers was confirmed by BLAST searches (GenBank database). A standard melting curve cycle was used to verify the quality of amplification and absence of primer dimer formation. The nucleotide sequences of ADAMTS-7 and GADPH primers are listed in Table 2. The relative expression levels of ADAMTS-7 in each sample were analyzed using the 2^−ΔΔCT^ method.

### 2.4. Protein Extraction and Western Blotting for ADAMTS-7, Full-Length COMP, and COMP Fragments

Tissue samples were conserved in liquid nitrogen; total protein was extracted and determined using a BCA Protein Assay Kit according to the manufacturer's instruction. Equal amounts of protein (10 *μ*g) for each sample were resolved by 10% acrylamide-SDS-PAGE. The samples were subsequently transferred to polyvinylidene difluoride membranes (PVDF; Millipore, Billerica, MA). Primary antibodies (rabbit anti-ADAMTS-7, 1 : 3,000, Abcam Biotechnology Co., Ltd., London, UK; rabbit anti-COMP, 1 : 3,000, Abcam Biotechnology Co., Ltd., London, UK), and a goat anti-rabbit immunoglobulin- (IgG-) horseradish peroxidase (HRP) secondary antibody (1 : 2,500; Beijing Golden Bridge Biotechnology Co., Ltd., Beijing, China) were applied. Equal amounts of protein loading were confirmed by reprobing the membranes with the rabbit anti-GAPDH-HRP antibody (1 : 5,000, Abcam Biotechnology Co.). Protein bands were detected using a FluorChem E Chemiluminescent Western Blot Imaging System (Cell Biosciences, Santa Clara, CA) and quantified by densitometry analysis using ImageJ software (National Institutes of Health, USA).

### 2.5. Statistical Analysis

All the data are presented as means ± SEM. Statistical analysis of the data was performed using unpaired Student's *t*-test and two-way analysis of variance (ANOVA) with Prism software followed by the Student-Newman-Keuls post hoc test. Differences were considered to be statistically significant if the *P* value < 0.05 (^*^ or ^#^) or 0.01 (^**^ or ^##^).

## 3. Results

### 3.1. Cartilage Degradation in the Pathogenesis of ONFH

We used H&E and safranin-O staining to determine the cartilage degradation in ONFH patients; Mankin score was also evaluated. The cartilages of FNF patients had mild moderated perichondrium, and the cells organizations were normal or mildly irregular with little chondrocyte clusters and necrosis; the safranin-O staining was normal or mildly reduced with intact tidemark. Groups II and III exhibit moderate-marked irregularity and more chondrocyte clusters and necrosis; the safranin-O staining of group II showed moderate-marked reduction while group III was almost not stained; their tidemark integrity was destroyed (Figure 1). We measured the Mankin score, and groups II and III exhibited significantly higher score than group I, in which group III got the highest (Figure 1(d)). The results indicated that, during the pathogenesis of ONFH, the cartilage has a progressive degradation.

### 3.2. Increased Expression of ADAMTS-7 and TNF-*α* in the Disease Course of ONFH

**Figure 2:**
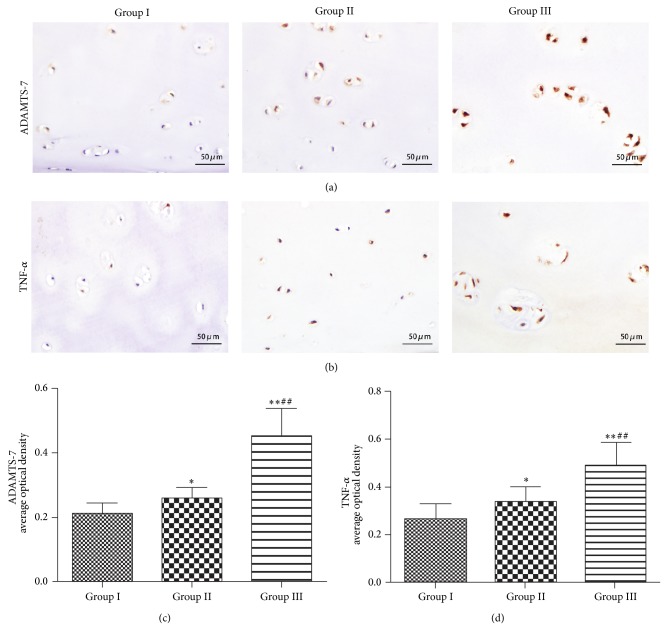
ADAMTS-7 and TNF-*α* are upregulated in ONFH patients. (a) Expression of ADAMTS-7 of cartilage samples of FNF patients and ONFH patients at different stages, assayed by immunohistochemistry. (b) Expression of TNF-*α* in different groups, examined by immunohistochemistry with anti-TNF-*α* serum. (c) Quantitative analysis of ADAMTS-7-positive area of each group. (d) Quantitative analysis of TNF-*α* area of each group. Statistically significant increases in the expression of ADAMTS-7 and TNF-*α* were observed in group II and group III compared with group I (^*^ *P* < 0.05, ^**^ *P* < 0.01). Significant increases in the ADAMTS-7 and TNF-*α* expression were observed in group III compared with group II (^#^ *P* < 0.05, ^##^ *P* < 0.01).

The immunohistochemistry results revealed that ADAMTS-7 and TNF-*α* were expressed in all three groups, but the levels of ADAMTS-7 and TNF-*α* were significantly increased in degraded cartilage of groups II and III. Furthermore, we also found a statistically significant increase in the expression of ADAMTS-7 and TNF-*α* in group III compared to in group II (Figure 2). The results suggested that the level of ADAMTS-7 was markedly upregulated with the degradation of cartilage.

### 3.3. Elevation of ADAMTS-7 Transcription Process in Degraded Cartilage

**Figure 3:**
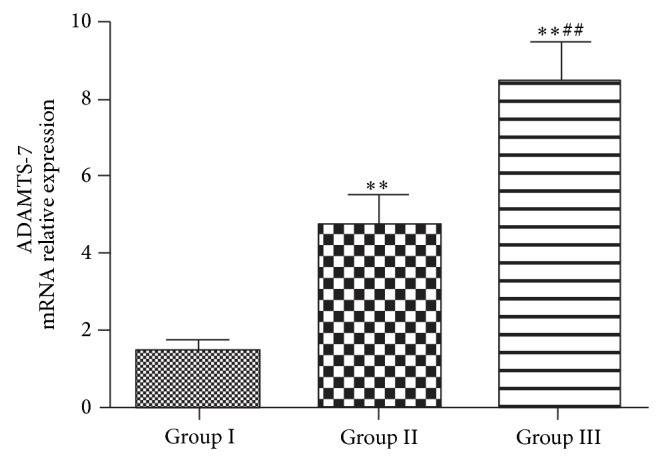
ADAMTS-7 mRNA expression in femur articular cartilage samples of each group: statistically significant increases in the mRNA expression of ADAMTS-7 were observed in group II and group III compared with group I (^*^ *P* < 0.05, ^**^ *P* < 0.01). A significant increase in the mRNA expression was observed in group III compared with group II (^#^ *P* < 0.05, ^##^ *P* < 0.01).

ADAMTS-7 mRNA is expressed in both degraded cartilage from ONFH patients and relatively normal cartilage from FNF patients. When we compared the expression levels of the target gene, a statistically significant increase in the gene expression of ADAMTS-7 was obtained in degraded cartilage compared with the relatively normal cartilage (Figure 3). What is more, in different groups of the degraded cartilage, there was also a significant increase in the ADAMTS-7 expression in cartilage of hip joint at stage 4 compared with that at stage 2-3.

### 3.4. Elevated ADAMTS-7 Level and Increased COMP Degradation in ONFH Patients

**Figure 4:**
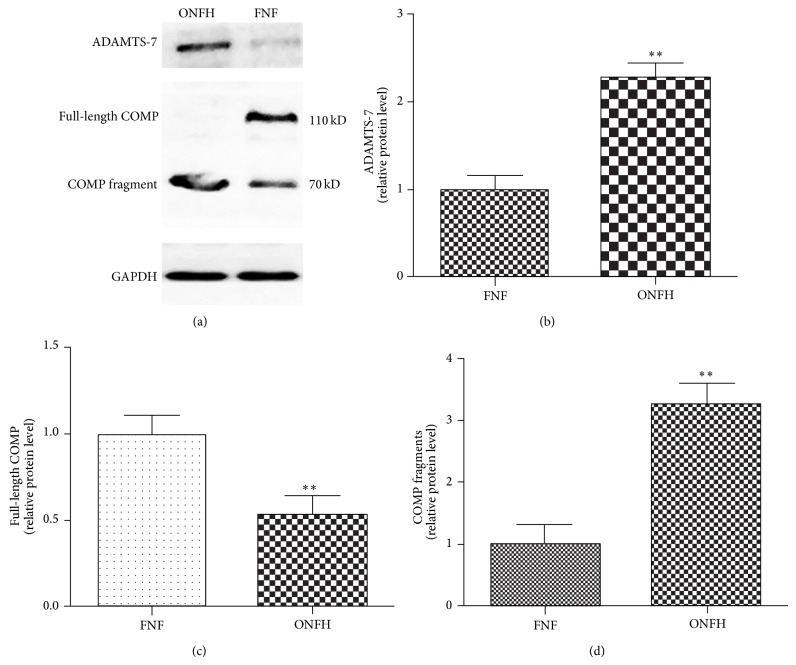
ADAMTS-7 associates with and degrades COMP in ONFH patients. (a) Representative western blotting of cartilage from ONFH and FNF patients. ADAMTS-7 (180 kDa), full-length COMP (110 kDa), and COMP fragment (≈65 kDa) were detected by western blot analysis. Expression levels of each gene are expressed as percentage of control. Expression level of ADAMTS-7 (b) and COMP fragment (d) is significantly increased in ONFH patients compared to in FNF patients, while the full-length COMP (c) level was decreased in ONFH patients (^*^ *P* < 0.05, ^**^ *P* < 0.01).

ADAMTS-7 has been proved to degrade COMP in OA and some circulation diseases [34–36]. So we examined the ADAMTS-7 and COMP protein level in FNF and ONFH patients' cartilage samples. Western blot analysis verified the significantly increased ADAMTS-7 protein level in ONFH patients. The COMP degradation was also observed in ONFH cartilage. With the increase of ADAMTS-7, full-length COMP decreased and COMP fragment accumulated (Figure 4). The result indicates a positive association between increased ADAMTS-7 and COMP degradation in ONFH patients.

### 3.5. Increased Expression of Phospho-NF-*κ*B P65 in Cartilage of ONFH Patients

**Figure 5:**
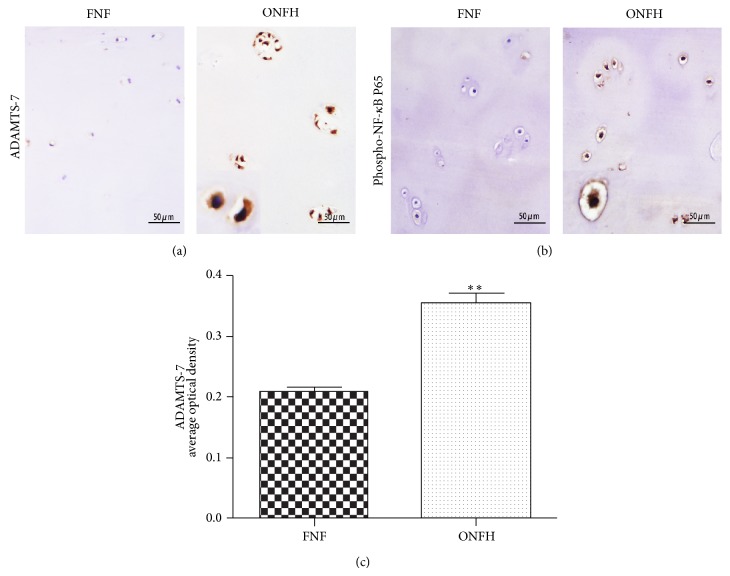
ADAMTS-7 and Phospho-NF-*κ*B P65 are upregulated in ONFH patients compared to FNF patients. (a) ADAMTS-7 expression of articular cartilage of FNF patients and ONFH patients, assayed by immunohistochemistry. (b) Phospho-NF-*κ*B expression in FNF group and ONFH group, examined by immunohistochemistry. The ONFH group exhibits high expression of Phospho-NF-*κ*B, and the FNF articular cartilage shows rare Phospho-NF-*κ*B expression. (c) Quantitative analysis of ADAMTS-7 area of FNF and ONFH group by average optical density (^*^ *P* < 0.05, ^**^ *P* < 0.01).

In order to investigate the mechanisms involved in the increased expression of ADAMTS-7 in cartilage of ONFH patients, we stained Phospho-NF-*κ*B P65 in the cartilage of both ONFH and FNF patients. The results showed that the ONFH groups exhibited significantly higher expression of Phospho-NF-*κ*B P65 compared with the FNF group, while Phospho-NF-*κ*B P65 was rarely expressed in relatively normal cartilage (Figure 5). Moreover, the ADAMTS-7 and Phospho-NF-*κ*B P65 are expressed synchronously in degraded and relatively normal cartilage.

## 4. Discussion

It has been proved that ADAMTS-7 plays an important role in the pathogenesis of OA [28]; it directly associates with and degrades COMP in vitro, and the level of ADAMTS-7 is elevated in cartilage patients with arthritis [34]. The cartilage of patients with ONFH exhibits a degraded condition with a relatively femoral head bone formative condition concurrently, which is different from patients with other hip diseases [37]. In the present study, we first evaluated the cartilage degradation levels of ONFH and FNF patients by Mankin score and examined the expression pattern of ADAMTS-7. Our results suggested that ADAMTS-7 expression level and Mankin score had comparable trend, which prompted us to speculate whether the ADAMTS-7 expression was significantly increased with the degradation of cartilage (Figures 1 and 2). It has been reported that cartilage specific overexpression results in cartilage degradation [38]. One possible explanation is that, in ONFH patients, the cartilage degradation is also related to ADAMTS-7.

In all three groups, the ONFH groups demonstrated an elevated level of ADAMTS-7 compared to the FNF group. In the comparison between the different stages of ONFH, the levels of ADAMTS-7 expression in the patients with ONFH changes (stage 4) were significantly increased compared with those observed in stage 2 and stage 3 patients (Figure 2). In the stage 4 ONFH group, which performed secondary osteoarthritis caused by ONFH, level of ADAMTS-7 was the highest among the stages. Similarly, RT-PCR with specific primers for human ADAMTS-7 showed that ADAMTS-7 mRNA level exhibits a parallel trend as the immunohistochemistry results, which is in line with ADAMTS-7 expression pattern in primary osteoarthritis [27]. On the other hand, the ADAMTS-7 expression level was enhanced just before the secondary osteoarthritis was formed or the femoral head collapsed, which indicates cartilage degradation throughout the whole process of ONFH pathogenesis and that ADAMTS-7 participated in cartilage degradation even in the early stage of ONFH. Taken together, the data implied that ADAMTS-7 might be involved in the cartilage pathogenesis of ONFH.

In the current study, we examined the COMP and ADAMTS-7 levels in FNF and ONFH patients. COMP is a prominent component of cartilage extracellular matrix (ECM); loss of COMP may lead to susceptibility of other cartilage matrix proteins, including Col II and aggrecan, and cause the degradation of cartilage. We revealed a parallel alternation between COMP degradation and increased ADAMTS-7 (Figure 4). It is reasonable to assume that cartilage degradation in ONFH depends, at least in part, on COMP degradation; what is more, ADAMTS-7 upregulation greatly enhanced COMP degradation in ONFH patients, which may strongly contribute to cartilage degradation in ONFH. Collectively, in the pathogenesis of ONFH, cartilage degradation may stem from COMP degradation induced by ADAMTS-7.

TNF-*α* is a well-described mediator of the inflammatory response and is synthesized and released in many diseases including OA. In the previous studies, TNF-*α* has been reported to play a crucial role in cartilage destruction in arthritis [39, 40]. The increased expression of ADAMTS-7 in the joint tissues of degenerative diseases, such as OA and rheumatoid arthritis (RA), patients is due to proinflammatory cytokines, including TNF-*α* [31, 41]. TNF-*α* enhances MMPs and ADAMTS expressions which could in turn cause exaggerated cartilage matrix degradation, while ADAMTS-7 also upregulated TNF-*α* expression in vitro and in vivo; it is indicated that ADAMTS-7 and TNF-*α* form a positive feedback regulatory loop in cartilage [31]. Our immunohistochemistry results showed that the TNF-*α* expression exhibited a concurrent increase with the increased expression of ADAMTS-7 in the radiographic groups (Figure 2). On the basis of the present study and the literature, our results suggest that at a late stage both primary OA and ONFH process a comparable inflammation status for the expression profile of TNF-*α*, major inflammatory cytokine, is similar. It also proved the concept that TNF-*α* can enhance ADAMTS-7 expression in ONFH patients and indicated that TNF-*α* also played an important role in the pathogenesis of ONFH. It has been reported that the hip labrum and synovium of the hip joint of ONFH patients exhibited less synovitis, less TNF expression than that of OA [26]. It is believed that time is a key factor in these differences. Our results showed that the expression of TNF-*α* was enhanced in cartilage of ONFH patients. In contrast, the traumatic injury of femoral neck fracture has a relatively short time process that simply does not facilitate the synthesis of TNF-*α*.

NF-*κ*B is a critical transcriptional regulatory factor which has been extensively investigated. It was reported that activity of NF is elevated during ONFH [42]. NF-*κ*B is activated and enters the cell nucleus and then it combines with specific DNA motifs and stimulates the expression of corresponding gene if the cells are stimulated by various cytokines. TNF-*α*-mediated activation of the NF-*κ*B signaling pathway plays an important role in the pathogenesis of OA [43]. What is more, the upregulation of ADAMTS-7 mediated by TNF-*α* is primarily through the NF-*κ*B signaling pathway [31]. Our results showed that the level of Phospho-NF-*κ*B P65 expression in ONFH patients was much higher than in femoral neck fracture patients (Figure 5). The NF-*κ*B pathway was activated in ONFH pathogenesis. It is interesting to surmise that TNF-*α* upregulates ADAMTS-7 expression throughout NF-*κ*B pathway in cartilage degradation of ONFH patients.

In conclusion, this study suggests that ADAMTS-7 is associated with and might be involved in cartilage degradation during the pathogenesis of ONFH, and its expression appears to interact with TNF-*α* mediated by NF-*κ*B. The detailed underlying mechanisms of these functions are still unknown, and subsequent studies are required to explore the related molecular interactions and signaling pathways.

## 5. Conclusion

Level of ADAMTS-7 is elevated in articular cartilage of osteonecrosis of femoral head; ADMATS-7 is positively associated with TNF-*α* and NF-*κ*B.

## Figures and Tables

**Table 1:** Mankin histological grading system.

Category	Grade
(1) Structure	
(a) Normal	0
(b) Surface irregularity	1
(c) Pannus and surface irregularity	2
(d) Clefts to transitional zone	3
(e) Clefts to radial zone	4
(f) Clefts to calcified zone	5
(g) Complete disorganization	6
(2) Cells	
(a) Normal	0
(b) Diffuse hypercellularity	1
(c) Cloning	2
(d) Hypocellularity	3
(3) Safranin-O staining	
(a) Normal	0
(b) Slight reduction	1
(c) Modest reduction	2
(d) Severe reduction	3
(e) No dye noted	4
(4) Tidemark integrity	
(a) Intact	0
(b) Crossed by blood vessels	1

**Table 2:** Real-time PCR primers.

Target	Forward primer, 5′-3′	Reverse primer, 5′-3′
GAPDH	GCACCGTCAAGGCTGAGAAC	TGGTGAAGACGCCAGTGGA
ADAMTS-7	GCAGGTTGAGAGCTATGTGCT	GCATGGTGCGTGATCTTTAGG

## References

[1] Cruess R. L., Ross D., Crawshaw E. (1975). The etiology of steroid induced avascular necrosis of bone. A laboratory and clinical study. *Clinical Orthopaedics and Related Research*.

[2] Hungerford D. S., Zizic T. M. (1978). Alcoholism associated ischemic necrosis of the femoral head. Early diagnosis and treatment. *Clinical Orthopaedics and Related Research*.

[3] Casey B. H., Hamilton H. W., Bobechko W. P. (1972). Reduction of acutely slipped upper femoral epiphysis. *Journal of Bone and Joint Surgery*.

[4] Csuka M., Brewer B. J., Lynch K. L., McCarty D. J. (1987). Osteonecrosis, fractures, and protrusio acetabuli secondary to X-irradiation therapy for prostatic carcinoma. *The Journal of Rheumatology*.

[5] Iwegbu C. G., Fleming A. F. (1985). Avascular necrosis of the femoral head in sickle-cell disease. A series from the Guinea Savannah of Nigeria. *Journal of Bone and Joint Surgery*.

[6] Amstutz H. C., Carey E. J. (1966). Skeletal manifestations and treatment of Gaucher's disease. Review of twenty cases. *The Journal of Bone and Joint Surgery. American*.

[7] Shinoda S., Hasegawa Y., Kawasaki S., Tagawa N., Iwata H. (1997). Magnetic resonance imaging of osteonecrosis in divers: comparison with plain radiographs. *Skeletal Radiology*.

[8] Jingushi S., Lohmander L. S., Shinmei M. (2000). Markers of joint tissue turnover in joint fluids from hips with osteonecrosis of the femoral head. *Journal of Orthopaedic Research*.

[9] Zalavras C. G., Lieberman J. R. (2014). Osteonecrosis of the femoral head: evaluation and treatment. *The Journal of the American Academy of Orthopaedic Surgeons*.

[10] Chandler F. A. (2001). Coronary disease of the hip. 1949. *Clinical Orthopaedics and Related Research*.

[11] Arlet J. (1992). Nontraumatic avascular necrosis of the femoral head: Past, present, and future. *Clinical Orthopaedics and Related Research*.

[12] Chen W.-L., Lin C.-T., Yao C.-C. (2006). In-vitro effects of dexamethasone on cellular proliferation, apoptosis, and Na+-K+-ATPase activity of bovine corneal endothelial cells. *Ocular Immunology and Inflammation*.

[13] Kerachian M. A., Harvey E. J., Cournoyer D., Chow T. Y. K., Séguin C. (2006). Avascular necrosis of the femoral head: vascular hypotheses. *Endothelium*.

[14] Hong J. M., Kim T. H., Kim H. J., Park E. K., Yang E. K., Kim S. Y. (2010). Genetic association of angiogenesis- and hypoxia-related gene polymorphisms with osteonecrosis of the femoral head. *Experimental and Molecular Medicine*.

[15] Zhang C., Yang F., Cornelia R., Tang W., Swisher S., Kim H. (2011). Hypoxia-inducible factor-1 is a positive regulator of Sox9 activity in femoral head osteonecrosis. *Bone*.

[16] Ficat R. P. (1985). Idiopathic bone necrosis of the femoral head. Early diagnosis and treatment. *Journal of Bone and Joint Surgery—Series B*.

[17] Grässel S., Beckmann J., Rath B., Vogel M., Grifka J., Tingart M. (2010). Expression profile of matrix metalloproteinase-2 and -9 and their endogenous tissue inhibitors in osteonecrotic femoral heads. *International Journal of Molecular Medicine*.

[18] Eckerwall G., Lohmander L. S., Wingstrand H. (1997). Increased levels of proteoglycan fragments and stromelysin in hip joint fluid in Legg-Calve-Perthes disease. *Journal of Pediatric Orthopaedics*.

[19] Hurskainen T. L., Hirohata S., Seldin M. F., Apte S. S. (1999). ADAM-TS5, ADAM-TS6, and ADAM-TS7, novel members of a new family of zinc metalloproteases. General features and genomic distribution of the ADAM-TS family. *The Journal of Biological Chemistry*.

[20] Barreda D. R., Hanington P. C., Walsh C. K., Wong P., Belosevic M. (2004). Differentially expressed genes that encode potential markers of goldfish macrophage development in vitro. *Developmental & Comparative Immunology*.

[21] Levy G. G., Nichols W. C., Lian E. C. (2001). Mutations in a member of the ADAMTS gene family cause thrombotic thrombocytopenic purpura. *Nature*.

[22] Roy R., Louis G., Loughlin K. R. (2008). Tumor-specific urinary matrix metalloproteinase fingerprinting: identification of high molecular weight urinary matrix metalloproteinase species. *Clinical Cancer Research*.

[23] Apte S. S. (2009). A disintegrin-like and metalloprotease (reprolysin-type) with thrombospondin type 1 motif (ADAMTS) superfamily: functions and mechanisms. *Journal of Biological Chemistry*.

[24] Fosang A. J., Little C. B. (2008). Drug insight: aggrecanases as therapeutic targets for osteoarthritis. *Nature Clinical Practice Rheumatology*.

[25] Murphy G., Nagase H. (2008). Reappraising metalloproteinases in rheumatoid arthritis and osteoarthritis: destruction or repair?. *Nature Clinical Practice Rheumatology*.

[26] Zhang Q., Huang M., Wang X., Xu X., Ni M., Wang Y. (2012). Negative effects of ADAMTS-7 and ADAMTS-12 on endplate cartilage differentiation. *Journal of Orthopaedic Research*.

[27] Guo F., Lai Y., Tian Q., Lin E. A., Kong L., Liu C. (2010). Granulin-epithelin precursor binds directly to ADAMTS-7 and ADAMTS-12 and inhibits their degradation of cartilage oligomeric matrix protein. *Arthritis and Rheumatism*.

[28] Liu C.-J. (2009). The role of ADAMTS-7 and ADAMTS-12 in the pathogenesis of arthritis. *Nature Clinical Practice Rheumatology*.

[29] Bai X. H., Wang D. W., Kong L. (2009). ADAMTS-7, a direct target of PTHrP, adversely regulates endochondral bone growth by associating with and inactivating GEP growth factor. *Molecular and Cellular Biology*.

[30] Luan Y., Kong L., Howell D. R. (2008). Inhibition of ADAMTS-7 and ADAMTS-12 degradation of cartilage oligomeric matrix protein by alpha-2-macroglobulin. *Osteoarthritis and Cartilage*.

[31] Lai Y., Bai X., Zhao Y. (2014). ADAMTS-7 forms a positive feedback loop with TNF-*α* in the pathogenesis of osteoarthritis. *Annals of the Rheumatic Diseases*.

[32] Steinberg M. E., Steinberg D. R. (2004). Classification systems for osteonecrosis: an overview. *Orthopedic Clinics of North America*.

[33] Mankin H. J., Johnson M. E., Lippiello L. (1981). Biochemical and metabolic abnormalities in articular cartilage from osteoarthritic human hips. III. Distribution and metabolism of amino sugar-containing macromolecules. *Journal of Bone and Joint Surgery*.

[34] Liu C. J., Kong W., Ilalov K. (2006). ADAMTS-7: a metalloproteinase that directly binds to and degrades cartilage oligomeric matrix protein. *The FASEB Journal*.

[35] Wang L., Zheng J., Bai X. (2009). ADAMTS-7 mediates vascular smooth muscle cell migration and neointima formation in balloon-injured rat arteries. *Circulation Research*.

[36] Du Y., Gao C., Liu Z. (2012). Upregulation of a disintegrin and metalloproteinase with thrombospondin motifs-7 by miR-29 repression mediates vascular smooth muscle calcification. *Arteriosclerosis, Thrombosis, and Vascular Biology*.

[37] Yamaguchi R., Yamamoto T., Motomura G. (2014). Bone and cartilage metabolism markers in synovial fluid of the hip joint with secondary osteoarthritis. *Rheumatology*.

[38] Neuhold L. A., Killar L., Zhao W. (2001). Postnatal expression in hyaline cartilage of constitutively active human collagenase-3 (MMP-13) induces osteoarthritis in mice. *The Journal of Clinical Investigation*.

[39] Furst D. E. (2010). Development of TNF inhibitor therapies for the treatment of rheumatoid arthritis. *Clinical and Experimental Rheumatology*.

[40] Tang W., Lu Y., Tian Q.-Y. (2011). The growth factor progranulin binds to tnf receptors and is therapeutic against inflammatory arthritis in mice. *Science*.

[41] Bevitt D. J., Mohamed J., Catterall J. B. (2003). Expression of ADAMTS metalloproteinases in the retinal pigment epithelium derived cell line ARPE-19: transcriptional regulation by TNF*α*. *Biochimica et Biophysica Acta—Gene Structure and Expression*.

[42] Tian L., Wen Q., Dang X., You W., Fan L., Wang K. (2014). Immune response associated with Toll-like receptor 4 signaling pathway leads to steroid-induced femoral head osteonecrosis. *BMC Musculoskeletal Disorders*.

[43] Kapoor M., Martel-Pelletier J., Lajeunesse D., Pelletier J.-P., Fahmi H. (2011). Role of proinflammatory cytokines in the pathophysiology of osteoarthritis. *Nature Reviews Rheumatology*.

